# Asian Pacific Endodontic Confederation (APEC) News 

**Published:** 2017

**Authors:** Mohsen Ramazani

**Affiliations:** a *APEC Councilor from Iran*

The 19^th^ meeting of Asian Pacific Endodontic Confederation (APEC) was held from 5^th^ till 8^th^ of April, 2017 at Hotel Hyatt Regency, New Delhi, India encompassing more than 1900 participants from 40 countries. The theme of the congress was "**Preserve to Stillness**"

*“An endodontist is the cardiac and neurosurgeon for the tooth. No other specialty involves as much focus, depth of involvement or may we say, a meditative state, as endodontics.*

*An endodontist’s ruminative endeavor is to bring the tooth to a state of “stillness.”*

*“Stillness” is rooted in the concept of “stillness meditation” where one relaxes the body to bring the mind to rest by itself. Simply “allows” it to happen.*

*Similarly, one allows the relationship of the periodontium, the occlusion and the functional ability of the tooth to become harmonious and structured to reach a state of stillness, where the tooth and its supporting structures are in accord with each other, by just performing endodontic treatment on the “troubled tooth”. “Preserve to stillness” is all we do.*

Worldwide respected speakers shared their knowledge and experience with the delegates. From Iran, Professor Abbasali Khademi and Dr. Mohammad Hossein Nekoofar as respectively the president and vice president of Iranian Association of Endodontists lectured. Also Dr. Saber Khazaei, PhD student of Endodontics and Dr. Mosleh Hamid, post graduate student of Pedodontics from Isfahan University of Medical Sciences accompanied us to have their oral and poster presentations.

Apart from scientific programs, the exhibition and social sections were also attractive.

A visit to India was not complete without taking advantage of discovering the rich culture, gastronomic traditions and multi colored folklore the country had provided. There were many opportunities to further discover India's biodiversity through specially selected excursions throughout the country.

At Biannual General Meeting of APEC 2017, the next office bearers were elected as follow:

***President*** **:** automatically Dr. Sanjay Miglani -India

***Immediate past president*** **:** automatically Dr. Gin Chen -Taiwan

***President Elect:*** Dr. Mehmet Baybora Kayahan -Turkey

***Secretary:*** Dr. Hyeon Cheol Henry Kim -South Korea

***Treasurer*** **:** James Brichko -Australia

***Educational Committee Chairman*** **:** Dr. Ibrahim Abu Tahun -Jordan

***Honorary Auditor:*** Professor Hideaki Suda -Japan and Professor Akbar Khayat -Iran.

Also it was approved that the APEC 2019 to be held in Istanbul, Turkey. The new official website of APEC was displayed as “www.apeconweb.org” 

The travel to beautiful India would be memorized forever, since apart from its own unique attractions the executive committee of APEC 2017 meeting particularly dear Dr. Sanjay Miglani did their best.

We hope that APEC would move toward the higher standards of scientific executions.

